# Remarks, Gleanings, and Extracts

**Published:** 1874-06

**Authors:** A. R. Kilpatrick

**Affiliations:** Texas


### REMARKS, GLEANINGS AND EXTRACTS

BY A. R. KILPATRICK, M.D., OF TEXAS.

*Diphtheria.*—In the April number, 1874, of the Charleston *Medical Journal and Review*, there is a very instructive article on *Diphtheria*, by Dr. Geo. E. Trescott. He contends that there is a difference between *croup* and *diphtheria;* and following the differentiation laid down by Dr. Stokes, he gives the following table:

| PRIMARY CROUP. | SECONDARY CROUP. |
|---|---|
| 1st. The air passages primarily engaged. | 1st. The laryngeal affection secondary to disease of *pharynx and mouth.* |
| 2d. The fever, symptomatic of the local disease. | 2d. The local disease arising in the course of another affection, which is generally accompanied by fever. |
| 3d. The fever inflammatory. | 3d. The fever typhoid. |
| 4th. The necessity for antiphlogistic treatment, and the frequent success of such treatment. | 4th. Incapability of bearing antiphlogistic treatment; necessity for tonic, revulsive and stimulating treatment. |
| 5th. The exudation spreading to the glottis from below upwards, the pharynx healthy. | 5th. The exudation spreading to the glottis from above downwards, the pharynx diseased. |
| 6th. The disease spasmodic, and in some situations epidemic, but never contagious. | 6th. The disease constantly epidemic and contagious. |

Dr. T. quotes from Bretonneau: "If the patient presents any evidence of disease of the nasal passages, a slight snuffing, or coryza during the prevalence of diphtheria, the finger should be placed behind the angle of the lower jaw, below the lobe of the ear, and thence passed down the side of the neck, and if swelling of the glands be noticed, it renders it probable that there is false membrane in the nares. If the upper lip is reddened only under the nostril corresponding to the side where the cervical glands are enlarged, it is quite certain there is diphtheria present."

*Contagion.*—Numerous authorities, such as Bard, Bretonneau, Trousseau, Niemeyer, Vogel, Guersent, Valleix, Rilliet, Barthez, Wood, Empis, and many others, contend it is contagious.

Does one attack exempt a person from a second? As diphtheria is a constitutional disease, in which the blood of the system is diseased in a manner similar to small-pox, scarlatina, etc., we may well infer that very few will have it a second time.

*Treatment.*—He depends chiefly on chlorate potassium, quinine, iron, and a general sustaining course. He seldom uses cauterants, and their employment is gradually being given up in England and Europe. Dr. Jacobi uses: ℞. Bromine, bromide pot., aa gr. i.; aqua, ℥vj. Apply to the throat.

Dr. Hertz recommends this wash for the throat: ℞. Carbolic acid crystals, ʒi.; alcohol, fl. ʒi.; tincture iodine, fl. ʒss.; aqua, fl. ʒv. Mix. Apply three or four times in twenty-four hours.

Others use chlorate and iodide pot. in combination with good effect.

He says a *distinct ulcer* rather points to some other disease than genuine diphtheria.

Dr. Trescott never calls a case diphtheria to laymen, unless the membrane forms.

In answer to the question, "Can we, by early and powerful medications, control the disease?" the answer generally is, we cannot. Dr. Marshall, of South Carolina, believes that by calomel and quinine, in large doses, during the first forty-eight hours, the disease can be greatly modified. Dr. Dean, of South Carolina, an old practitioner, has seen cases where the early administration of an emetic has had a most decided effect in saving the patient. Dr. Hoke, of South Carolina, who has had a large experience, does not believe that in genuine diphtheria we possess any remedy by which we can control the disease. Dr. Hoke says, in one fatal case, he carefully removed, with the forceps and scissors, the entire membrane, and in three hours by the watch it had reformed, and appeared to be even thicker than before.

In an able and very interesting article on *Diphtheria*, by Dr. Benjamin Lee, read before the Philadelphia County Medical Society, 11th February, 1874, and published in the *Medical Times*, 16th May, he takes the opposite ground, that this disease and *croup* are the same. He properly criticizes, and objects to, the name *diphtheritis*, as being a misnomer; meaning, literally, an inflammation of a false membrane.

He quotes, in proof of his opinion, the writings on this disease of Dr. Max Jaffe, of Hamburg; Dr. Oertel, of Munich; Dr. Ludwig Letzerich, of Vienna; Dr. Conrad Küster, of Berlin; Dr. Lewin, of Berlin; Dr. Franz Hartman, of Wiesbaden, and Dr. Morell McKenzie, of Great Britain—all of whom say, in substance, that the distinction between diphtheria and croup was one which could no longer be maintained. That neither is always, and both are sometimes, epidemic and contagious; that the exudation is essentially the same, being modified by its site, but presenting histologically no marked difference—that of dipththeria having been noticed to become organized as well as that of croup; and that the sequelæ of diphtheria—albuminuria and impaired innervation—have also been observed to follow croup.

Of course, Dr. Lee contends for a supporting and tonic course of treatment, together with remedies to counteract the systemic poison.

A favorite remedy is the following: Quinine freely, and—℞. Potas. chlorate, ℈ viij.; acid hydrochloric, m. viij. Rub together until greenish fumes of chlorine begin to rise, then add aqua cinnamon, fl. ℥ viij. M. S. A tablespoonful every two hours.

He says Dr. Fordyce Barker's favorite remedy is *Turpeth Mineral*, or yellow sulphate of mercury. It acts as an emetic more promptly and efficiently than alum or ipecac; it is tasteless, does not exhaust and depress the vital power; it is equally prompt as sulphate of copper, and much more effective as a sedative and revulsive.

*Cerebro-Spinal Meningitis.*—Dr. Wm. Read, of Boston, Mass., published in Nos. 20 and 21, May, 1874, of Philadelphia *Medical and Surgical Reporter* the treatment of twenty cases of this disease in Boston City Hospital in 1873 and 1874. The details of symptoms, changes, treatment, etc., are full, precise and interesting. Some of the cases, if they had occurred in a Southern section, or in the practice of a less observant physician, would not have been classed as meningeal, or been treated as they were by him. He acknowledges his indebtedness to Dr. Brown-Sequard for the suggestion of ergot and belladonna as remedial agents in this disease. Ergotin, *one grain;* ext. belladonna, $\frac{1}{10}$ gr.; repeated every three hours. This diminishes the amount of blood supplied to the brain and spinal cord. In only one case was any relaxation of the iris observed. It seems to be a characteristic feature, or symptom, that the pupil does not enlarge from the use of belladonna in this disease. He used many other medicines, as they were called for by the necessities of the cases, such as cathartics, diaphoretics, stimulants, cups, blisters, etc. Out of the whole number only three died under his treatment.

The writer now has a case of the kind under treatment, and has used atropia by the endermic syringe, and has given belladonna internally, and has observed closely, and has at no time seen any dilatation of the pupils. The case has been very severe; hemiplegia at first, delirium, hyperosthesia, all of which are gradually and steadily yielding.

*Action of Cold Water on the Spleen.*—Dr. F. Mosler has arrived at the following conclusions from experiments on the action of water on the exposed spleens of animals: 1. The immediate contact of water with the normal spleen produces a visible contraction of the organ, varying in degree with the temperature of the water and the duration of the application. 2. In a less degree, cold water exerts the same action on the spleen through the intestinal walls. The effect of a cold douche is greater than that of the application of cold compresses or pieces of ice; probably the mechanical influence plays a part here. The action of water is inferior to that of quinia in causing contraction of the spleen. 3. Cold water also produces diminution in the size of splenic tumors, both acute and chronic. 4. The febrile paroxysms in ague may be arrested by cold douches applied after Fleury's method. 5. The cold douche does not supersede the use of quinia either in recent or in chronic cases of intermittent. 6. The therapeutic action of the cold douche in intermittent fever is not complete. It does not prevent relapses nor the formation of splenic tumors. 7. The splenic tumor in typhus is reduced in size by the use of cold water. 8. Much good is to be expected from a combination of the application of cold over the spleen, either in the form of ice or of the cold douche, with the administration of quinia.—*Baltimore Physician and Surgeon.*

*Chloral Hydrate Successfully used in Tetanus.*—Dr. Coryllos, of Patras in Greece, published a case of this kind in the *Allgem. Wiener Med. Zeit.*, No. 2, 1873, and now the same author records two similar cases, one under his own care, and the other treated by Dr. Basilin. The latter case relates to a woman of forty, who had wounded her finger with a splinter, which she removed herself. Tetanus occurred one month after the accident, and she had more than ten general attacks in the twenty-four hours. Sixteen days after the first tetanic symptoms the patient removed from the wound a bit of splinter, the size of a pin, which had been left in it unobserved. The usual narcotics having failed, chloral was tried and succeeded. Altogether three ounces and a half were taken in twenty days.

In Dr. Coryllos' case a man of forty had his left temple wounded by a pointed piece of reed. Tetanus supervened, and here, again, a portion of the foreign body was removed twelve days after the accident. He had at first fifteen-grain doses of chloral, and improved much upon them, but the tetanus recurred with renewed severity, and the chloral was pushed as high as 120 grains per diem. The patient recovered, and had taken, in about thirty days, six ounces of chloral.—*Lancet*, April 4, 1874.

*A Vegetable Cure for Syphilis.*.—C. C. Graham, M.D., (*American Practitioner*) says: "Having lived my first century pretty well out, and thinking that I may sometime or other die, I desire through your journal to make known to the medical public a discovery I made many years ago, of a vegetable cure for syphilis. This is a mixture of datura stramonium and phytolacca decandra. Of the best proportions of the ingredients I am not now able to speak with confidence, but my recollection is that I made the tincture of one ounce of the stramonium seed and half a pound of the poke-root to two quarts of common whisky. I am sure I have in fifty years treated by this tincture twenty cases of syphilis, many of them of the worst form, with perfect success. It is proper to add that I got the hint of poke-root as a remedy in the disease as long ago as 1815, when I was a pupil in Dr. B. W. Dudley's office, at Lexington, where I saw it successfully given in the case of a negro man."

*How to Check Coughs.*—Dr. Brown-Sequard, in his late Boston Lectures, says that there are many facts which show that morbid phenomena of respiration can always be stopped by the influence of arrest. Coughing, for instance, can be stopped by pressing on the nerves of the lip in the neighborhood of the nose. A pressure there may prevent a cough when it is beginning. Sneezing may be stopped by the same mechanism. Pressing in the neighborhood of the ear, right in front of the ear, may stop coughing. It is also preventive of hiccough, but much less so than of sneezing or coughing. Pressing very hard on the top of the mouth inside is also a means of stopping coughing. And he adds that the will has immense power there. There was a French nurse who used to say, "The first patient who coughs here will be deprived of his food to-day." It was exceedingly rare that a patient coughed then.—*Med. and Surg. Reporter*, April, 1874.

*Whooping-Cough.*—Dr. Stevens, of Ilminster, gives his experience with various remedies in this disease, in the *British Medical Journal*, as follows:

I must give the preference, in an ordinary case, to small doses of compound tincture of benzoin, frequently repeated. If the cough be more than usually spasmodic, I find dilute hydrocyanic acid, combined with bromide of potassium and camphor mixture, very serviceable; in the latter stage of the disease I much prefer alum, combined with dilute nitric acid and gentian, to any other astringent tonic; although in all cases everything depends upon the diathesis of the patient. I was greatly disappointed in the use of chloral hydrate, as in one case only could I detect the slightest benefit.—*Med. and Surg. Reporter*, April, 1874.

*Treatment of Diabetes.*—In a paper recently published (*Sitzungsberichte der Kais. Akad. d. Wiss. zu Wein*, Bd. lxvi. Heft iii., iv. and v.) Dr. Kratschmer gives the results of a series of observations he has made on a diabetic patient, with a view of testing the value of carbonate and sulphate of soda, and of morphia, upon the excretion of sugar. He finds that neither the carbonate nor the sulphate appeared to exert any influence on the amount of sugar excreted, but he has satisfied himself that in morphia we possess an agent that is not only capable of materially reducing the excretion of sugar, but also of diminishing to a remarkable extent the general tissue metamorphosis of the body.—*Practitioner*, April, 1874.

*Eczema Capitis.*—Cure it if possible, notwithstanding the somewhat prevalent idea to the contrary. The methed of procedure recommended is as follows:

First, apply a poultice every night until all the scabs are removed. The ulcerations, which are sometimes present after the scabs have been removed, are best cured by the application of a wash made of nitrate of silver grs. v. to the ℥i. of water. The following is then used with good success: ℞. Aquæ cologne, ℥iv.; glycerin, ℥ij.; carbolic acid crystals, ʒi.; borax, ʒi. M. Continue the application for some time for the purpose of curing the eczema.—*Medical Record*, December, 1873.

*Gelseminum in Gynæcologia.*—Dr. H. V. Ferrel, in the *Clinic*, says he has found gelseminum to possess the property of causing a dilatation of the os uteri.—*Archives of American Medicine and Surgery*, March, 1874.

---

